# Hybrid surgery of arteriovenous malformation and aneurysm of the sole

**DOI:** 10.1002/ccr3.7731

**Published:** 2023-07-30

**Authors:** Iraj Nazari, Seyyed Masoud Mousavi, Mohammad Amin Zargar, Seyed Mohammad Amin Alavi

**Affiliations:** ^1^ Department of General Surgery School of Medicine Ahvaz Jundishapur University of Medical Sciences Ahvaz Iran; ^2^ Faculty of Medicine Ahvaz Jundishapur University of Medical Sciences Ahvaz Iran

**Keywords:** aneurysm, angiography, arteriovenous malformations, embolotherapy, foot, surgery

## Abstract

The study describes the successful treatment of a rare type of arteriovenous malformation (AVM) in the sole using hybrid surgery, which consists of open resection and embolization. Moreover, the possibility of utilizing ultrasound during examination in addition to angiography for the diagnosing of AVM of the sole is proposed.

## 1 INTRODUCTION

The study describes the successful treatment of a rare type of arteriovenous malformation (AVM) in the sole using hybrid surgery, which consists of open resection and embolization. Moreover, the possibility of utilizing ultrasound during examination in addition to angiography for the diagnosing of AVM of the sole is proposed.^1^ AVMs can be found in different body parts but are most frequently found on the head, neck, and lower extremities.^2^ AVM of the sole is a rare disease that can impede normal activities.^3^ The first case report related to AVM of the sole was published in 1969. Since then, only a limited number of papers regarding AVM of the sole have been published^1^, ^3^, ^4^, ^5^, ^6^, ^7^, ^8^, ^9^ and the current research it is believed to be the ninth published article regarding AVM of the sole to date.

Herein, the authors have described a case of a 31‐year‐old female suffering from AVM of the sole who was treated via hybrid surgery (open resection and endovascular embolization).

## 2 CASE PRESENTATION

A 31‐year‐old female patient was presented to the Golestan Hospital, affiliated with Ahvaz Jundishapur University of Medical Sciences. The patient was suffering from an incrementally increasing severe pain in the sole of her right foot over a six‐month period since the referral date, especially during walking.

The patient's medical history was unremarkable, showing no history of surgery or trauma to the lower limbs. Physical examination showed no discoloration or skin lesions; however, a pulsating mass of 7 cm was felt behind the ankle. Multiple tender pulsatile masses up to 4 cm in diameter were also observed on the sole.

Ultrasound of the sole revealed an aneurysm in the posterior tibial artery in the ankle region, and numerous prominent vessels were observed in the sole.

**FIGURE 1:**
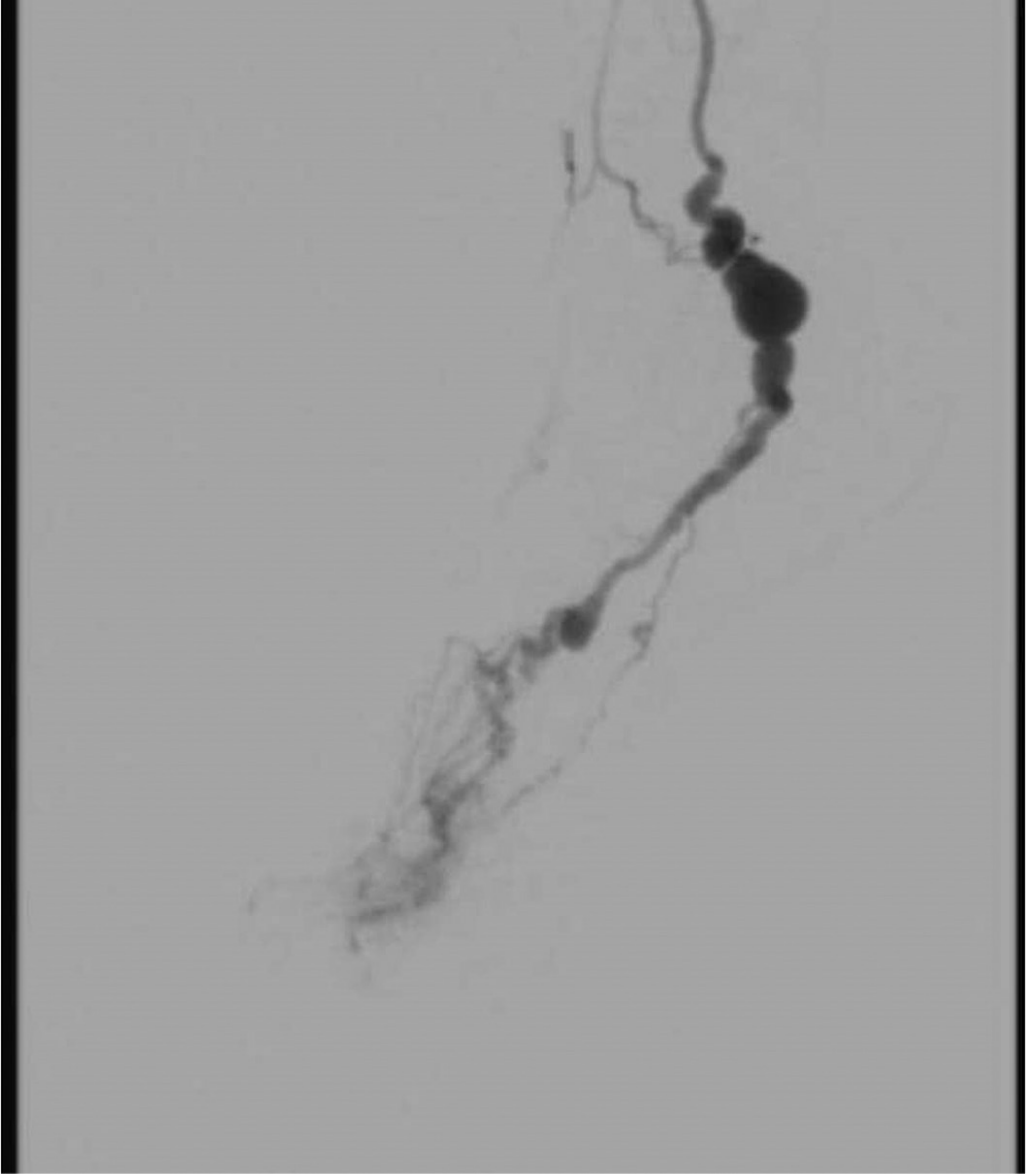
Pre‐operation angiography revealing the aneurysm in the posterior tibia artery behind the ankle, along with the arteriovenous malformation (AVM) of the sole.

The decision to perform a diagnostic angiography was based on the physical examination and ultrasound findings, which revealed an aneurysm in the posterior tibia artery behind the ankle, along with an AVM in the sole (Figure 1). Due to the location of the AVM and the need to remove the aneurysmal part of the posterior tibia artery, a hybrid surgery consisting of open resection and endovascular embolization was proposed.

**FIGURE 2:**
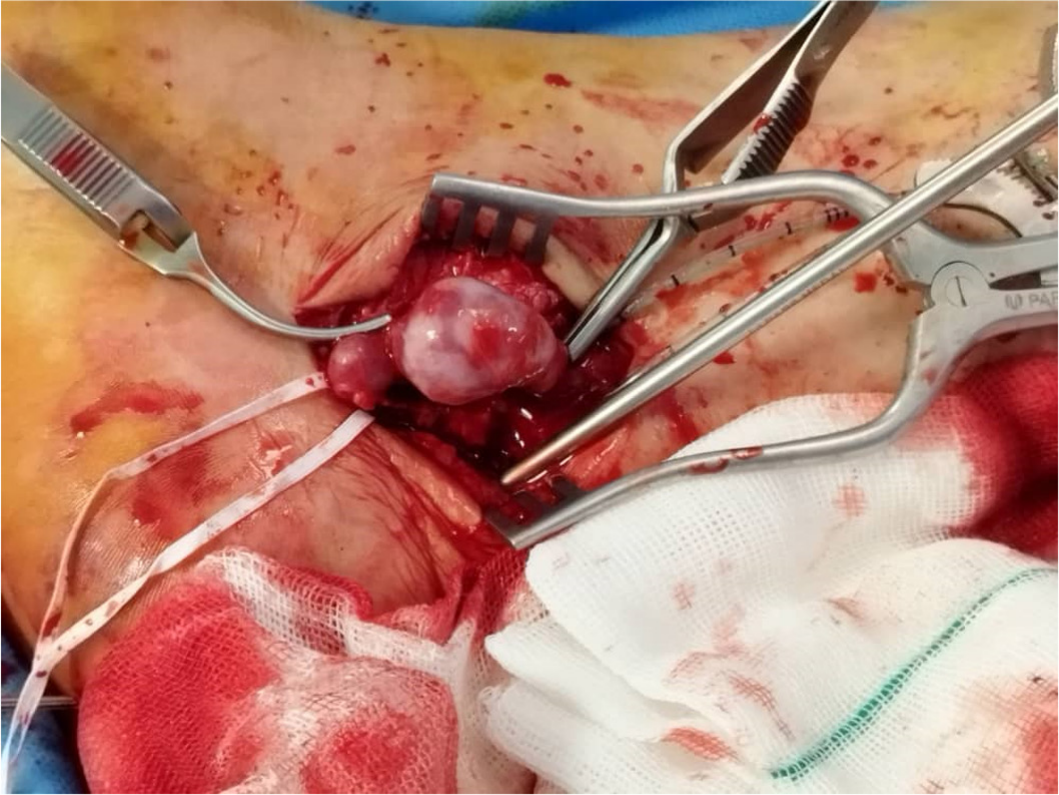
The aneurysmal part of the posterior tibia artery was carefully released, and proximal and distal aneurysms were controlled by tape.

Spinal anesthesia was applied to the patient, and the skin was incised across the posteromedial of the malleolus. The aneurysmal part of the posterior tibia artery was carefully released, and proximal and distal aneurysms were controlled using tape (Figure 2). Using a guide wire, a 5F series flat sheet membrane was inserted through the distal part of the aneurysm into the artery and fixed. Simultaneously, an angiography of the sole was performed to identify the feeding arteries of the AVM. First, the proximal section of the aneurysm was clamped, and then embolization was carried out using polyvinyl alcohol (PVA). Due to the large size of the AVM, gel foam embolization was also performed for the patient. Ultimately, the main feeding branch was closed with a coil.

**FIGURE 3:**
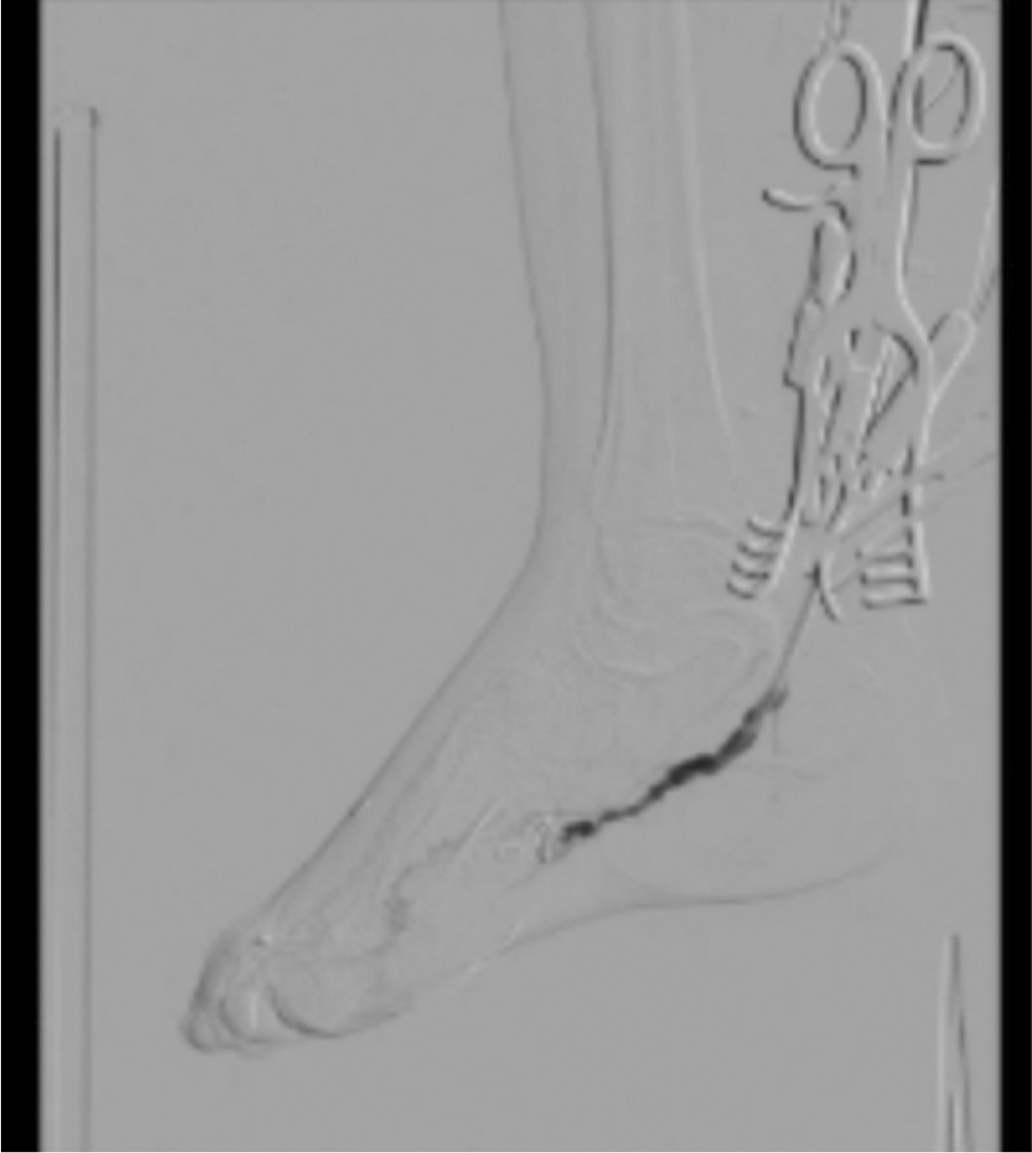
Post‐operation angiography revealing significant closure of the arteriovenous malformation (AVM).

Post‐operation angiography was then carried out, revealing that a significant part of the AVM was closed (Figure 3).

**FIGURE 4:**
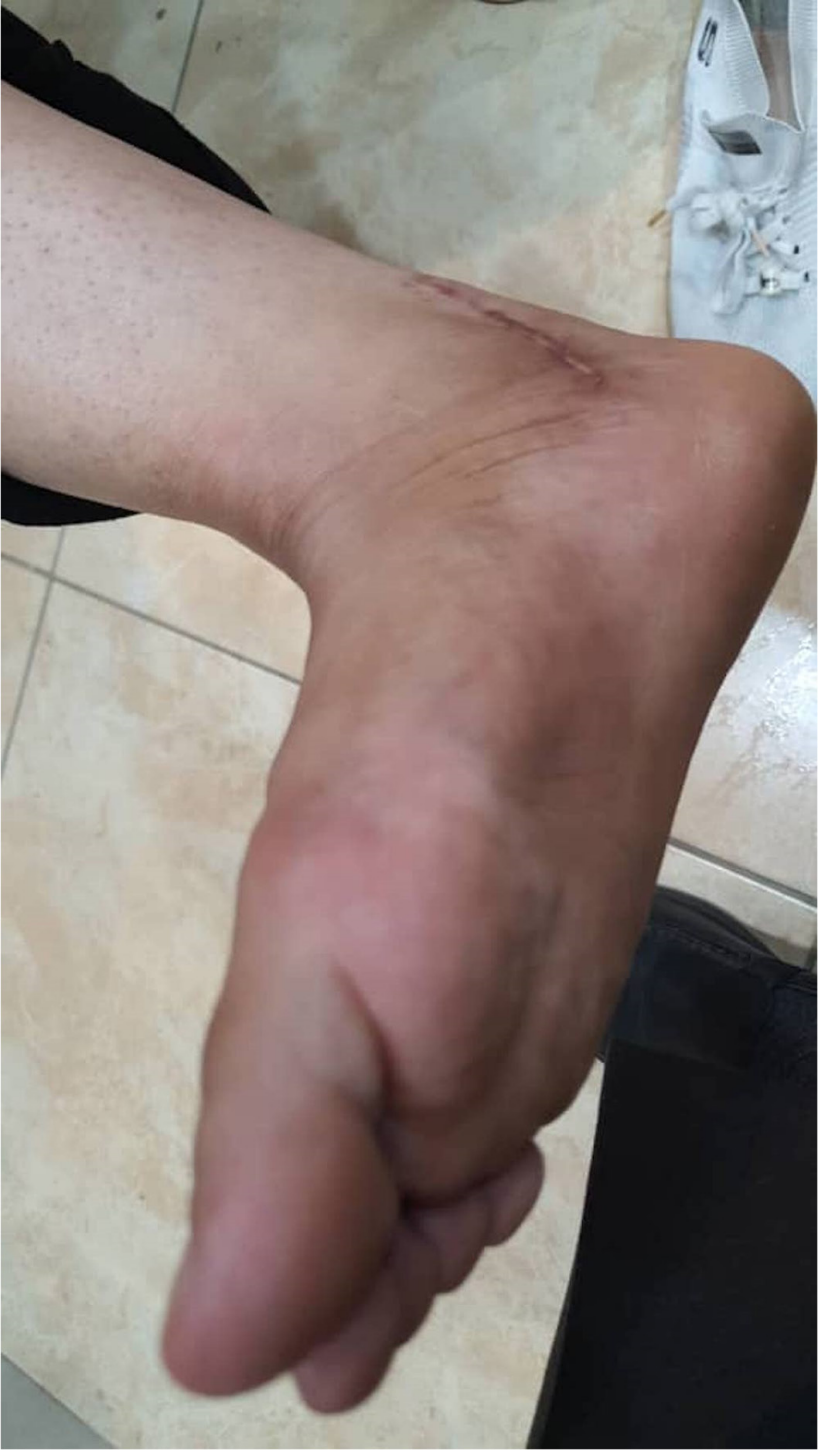
Evaluation after discharge.

After controlling the proximal and distal aneurysmal part of the posterior tibial artery, the aneurysmal section was removed. The saphenous vein of the same side was used to interpose the posterior tibial artery graft. After ensuring hemostasis, the skin was sutured, and the wound was bandaged. In the postoperative examination, the patient had no movement disorders, and the pulsating mass on the sole was not palpable. Following the patient's discharge, several evaluations were carried out at various periods, revealing that the patient had no subsequent problems putting her weight on her sole (Figure 4).

## 3 DISCUSSION

An AVM is a rare condition in as such that the incidence and prevalence of AVM are 1/100,000 and 10/100,000. The literature on the subject indicates that gender, race, and ethnicity do not increase the risk of AVM; moreover, AVMs are believed to result from abnormal differentiation of the vascular system throughout embryogenesis.^10^, ^11^

In most cases, symptoms begin between the ages of 35 and 40 but can occur at any time.^11^

While AVMs exist at birth, they are asymptomatic; however, during puberty and pregnancy, AVMs can become symptomatic due to the influence of hormones.^9^ An AVM presents signs and symptoms such as thrill and pulsation, a sensation of heat, port‐wine stain, pain, tissue bleeding, and necrosis.^12^ AVM of the foot could substantially affect the patient's movement and quality of life.^9^

The Solitary AVM in healthy individuals is considered an anomaly; however, multiple lesions are associated with congenital syndromes, including Klippel and Trenaunay, Sturge Weber, Parkes Weber, and Proteus syndromes.^9^

Noninvasive investigations such as ultrasound (US), computed tomography (CT), and magnetic resonance imaging (MRI) are frequently used to diagnose peripheral lesions; however, for diagnosing AVM, an invasive investigation using angiography can also be applied.^3^ While ultrasound shows multiple vascular channels with supplying arteries, nidus, and draining veins as multiple anechoic spaces,^13^ catheter angiography is the gold standard for identifying the primary blood supply to the vascular malformation, the existence of a nidus, the magnitude of arteriovenous shunting, and venous drainage.^14^

The primary treatment for AVM of the sole is surgical excision with ligation or embolization of the feeding vessels.^9^ The therapeutic target for AVMs is to eradicate the nidus in order to alleviate symptoms and prevent recurrence. Surgical excision in such a case is a difficult procedure as AVMs commonly develop within normal tissue where the irreversible loss of normal functioning is unavoidable.^3^ AVMs near joints, tendons, and ligaments could have an adverse impact on such tissues. In such a case, extensive surgical excision might expose the underlying soft tissue, requiring further treatment to produce a cover for the underlying soft tissue.^9^

Numerous agents, such as gel foam, coils, PVA particles, glue, and detachable balloons, have been utilized for embolization. Selecting the most suitable agent depends on two parameters: (1) the size of the vessel and (2) the temporary or permanent closure of the vessel.^15^, ^16^ Coil embolization of the feeding artery is a challenging and potentially dangerous procedure due to the formation of numerous collateral feeders that shift blood to the nidus.^3^ Embolization can alleviate symptoms within an approximate range of 40%–80% of the cases and cure the problem completely in 10% of patients; however, the recurrence rate of diffuse AVM in extremities is about 50%.^17^ The complications of applying any embolic agent vary from 10 to 30%, of which the most common complications are tissue necrosis and neuropathy.^8^ Adjunctive embolization before surgical resection could lead to lower incidences of hemorrhage and long‐term recurrence of the AVM while enhancing surgical outcomes.^18^

In the current investigation, the most critical issue was the aneurysm formation of the posterior tibial artery. The aneurysm was larger than normal and painful. Moreover, the aneurysm had the potential to rupture at any time. There was also a risk of aneurismal rupture in case of primary AVM surgery through retrograde angiography from the femoral artery. Due to the location of the aneurysm, which was behind the ankle, a stent graft procedure was impossible; therefore, the vascular surgery team decided to use a hybrid technique to simultaneously perform surgery on both the aneurysm and AVM.

## 4 CONCLUSION

The authors presented a rare AVM in the sole, for which hybrid surgery consisting of open resection and embolization was applied as the proposed treatment. Due to its rarity, most surgeons have seldom dealt with AVM of the sole during their routine practice, which in itself emphasizes the importance of considering AVM when treating chronic pain in the plantar region. A combination of ultrasound examination and angiography could also aid physicians in diagnosing AVM of the sole.

## AUTHOR CONTRIBUTIONS

**Iraj Nazari:** Writing – original draft; writing – review and editing. **Seyyed Masoud Mousavi:** Writing – original draft; writing – review and editing. **Mohammad Amin Zargar:** Writing – original draft; writing – review and editing. **Seyed Mohammad Amin Alavi:** Writing – original draft; writing – review and editing.

## FUNDING INFORMATION

No sources of funding were declared for this study.

## CONFLICT OF INTEREST STATEMENT

The authors have no conflict of interest to declare.

## ETHICS STATEMENT

Permission from the ethics committee was not required for case reports at the institution where the research was carried out.

## CONSENT

Written informed consent for publication of the case report has been signed by the patient and is available upon request from the editors.

## Data Availability

The data supporting the findings of this study are available upon request from the corresponding author.
